# Development of a Spectroscopic Map to Explain the Broad Raman Peak for Alkynes Solvated in Triethylamine

**DOI:** 10.1021/acs.jpcb.5c05298

**Published:** 2025-08-12

**Authors:** Anagha Aneesh, Kristina Streu, Clyde A. Daly

**Affiliations:** Department of Chemistry, 3776 Haverford College, 370 Lancaster Ave Haverford, Haverford, Pennsylvania 19041, United States

## Abstract

The terminal alkyne C≡C stretch has a large Raman scattering cross section in the “silent” region for biomolecules. Experimental work taking advantage of this property provide an impetus for the development of theoretical tools addressing the vibration. In prior work, we have developed a localized normal mode method for computing terminal alkyne vibrational frequencies using a discrete variable representation of the potential energy surface. Using this method and molecular dynamics simulations, we interpret the unusually broad Raman spectrum of alkynes solvated in triethylamine. Energy decomposition analysis is performed on alkyne-triethylamine dimers to determine that charge transfer, electrostatics, and Pauli repulsion have large effects on the frequency. Molecular dynamics simulations of triethylamine-solvated alkynes are performed and uncover that the terminal alkyne hydrogen interacts strongly with the triethylamine nitrogen. Interactions persist for 3–10 ps. Using this data, a spectroscopic map for terminal alkynes is developed and used to compute Raman spectra for alkynes in triethylamine. We find that the broad experimental spectra result from the combination of a population of alkynes associated with the solvent nitrogens and a population not associated with those nitrogens. This work sets the stage for investigations of alkynes in more complex environments like proteins and nanomaterial surfaces.

## Graphical abstract

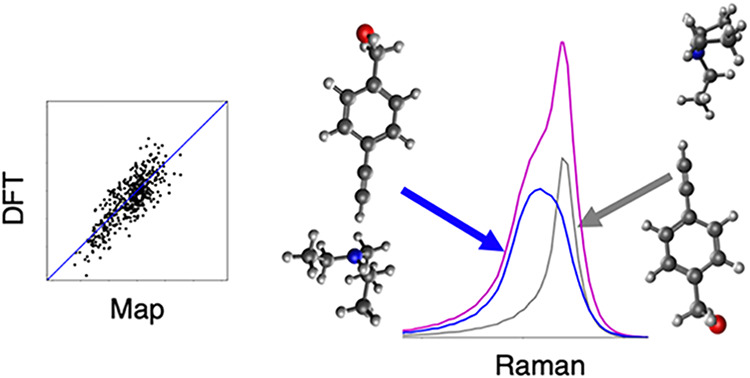

## Introduction

Infrared and Raman spectroscopy are effective tools for understanding the structure and dynamics of complex condensed phase systems.^1–3^ The interpretation of these spectra can be aided by the use of vibrational probes.^4–6^ A good probe exhibits reliable changes in its spectrum in response to specific changes in its environment. For example, OH and OD groups report on the propensity of the system to donate or accept hydrogen bonds. When donating a hydrogen bond, the OH or OD frequency decreases; when accepting a hydrogen bond, adjacent OH or OD frequencies increase.^7^ Terminal alkynes, which absorb weakly in the IR but have strong Raman signals, have recently been explored as possible vibrational probes.^8–12^ They have already been used as tags to investigate some biochemical problems due to their small size, biorthogonality, and strong peak in the transparent window of Raman spectra (1800–2600 cm^–1^).^13^ Tagging studies are focused on detecting the presence of the functional group and the object it is attached to. In contrast, probing studies seek to use differences in observed spectra to infer differences in environmental conditions in the vicinity of the probe. However, to use alkynes as a probe, the specific environmental factors that contribute to changes in frequency must be carefully determined, if there are any.

In a recent paper, Romei et al. experimentally determined the Raman scattering of two terminal alkyne molecules in a variety of solvents with the aim of developing the terminal alkyne as a new probe group.^8^ One of the most interesting results of this study was for triethylamine (TEA). The C≡C stretch Raman peaks for alkynes in this solvent were particularly wide and seemed to be made from two subpeaks. One subpeak had a significantly lower center frequency than the other. Raman spectra of the same alkynes in other solvents had no subpeaks, only single main peaks. However, the center frequencies of alkynes in some solvents were lower than either TEA-solvent subpeak, while center frequencies for alkynes in some other solvents were higher than either TEA-solvent subpeak. Roughly, solvents where alkynes had lower center frequencies tended to have more Lewis basic environments, and higher frequencies were related to electron poor environments (though there were significant exceptions to this rule). Tentatively, the researchers proposed that there are two subpopulations for alkynes in TEA – one where the alkyne is in an electron rich environment, and one where the alkyne is in an electron poor environment. However, the lack of clarity in these trends in the other solvents clouds this picture. We aim to clarify the origin of the subpeaks in the experimental Raman spectra of alkynes in TEA using computational chemistry methods.

In this work, we use density functional theory (DFT) calculations, molecular dynamics (MD) simulations, and our recently developed localized normal mode discrete variable representation (LNM-DVR) vibrational method to determine the molecular origin of these Raman peaks.^14^ We focus on the same molecules as were used in the experiments, 4-ethynylbenzyl alcohol and propargyl acetate. From MD, we find that the alkyne moiety can occupy two environments: one where it faces the nitrogen in TEA, and one where it faces the ethyl groups. It moves between these environments relatively slowly. From DFT, we find that the frequency changes substantially between these two environments and that a combination of electrostatics, charge transfer, and Pauli repulsion is the cause of most of this change. Finally, we extract snapshots from MD simulations of the alkynes in TEA and use LNM-DVR to calculate the contribution to the C≡C stretch vibrational frequency due to the solvent. We develop a terminal alkyne C≡C stretch spectroscopic map by fitting these frequencies to variables based on the electric field at the terminal alkyne hydrogen and the repulsive portion of the Lennard-Jones potential. Using this spectroscopic map, we compute Raman spectra. We find that these computed spectra are asymmetric in a similar way to the experiment. Overall, we are able to confirm that, at least in TEA, the alkyne vibration reports most strongly on the electron density of its environment.

## Methods

### Dimer Frequency Calculations

All density functional theory (DFT) calculations were performed in Q-Chem 5.4 using B3LYP-D3/def2-TZVPD, TPSS-D3/6–311++G**, or PBEh-3c/def2-mSVP.^15–20^ We investigated the interactions of two terminal alkyne molecules with triethylamine (TEA); these were 4-ethynylbenzyl alcohol (EBA) and propargyl acetate (PAC). Initial structures of these molecules interacting with a single TEA molecule were obtained from an MD simulation (described below). The dimer geometries were optimized to a maximum gradient of 3 × 10^–6^ atomic units. If this was not possible, the gradient was instead optimized to a maximum of 30 × 10^–6^ atomic units. We constrained the TEA nitrogen to terminal hydrogen distance to 20 evenly spaced values between about 2 Å (the optimized structure with no constraints) and about 11.65 Å (enough distance that the two molecules were no longer interacting). The structures were optimized under these distance constraints. The LNM-DVR method developed in previous work was used to compute the anharmonic frequency for the C≡C stretch vibration at each geometry.^14,21^ In this way, we obtained anharmonic normal-mode frequencies across an array of nitrogen–hydrogen distances. Based on our prior work, we used scaling factors of 0.989 (TPSS), 0.966 (B3LYP), and 0.931 (PBEh-3c) to correct inaccuracies in our LNM-DVR calculations. In our prior work, we were surprised to find that TPSS-D3/6–311++G** provided a great deal of accuracy for terminal alkyne vibrational calculations in the gas phase (notice the scaling factor is close to 1).^14^ While TPSS appears to model alkyne vibrations well, it may not be so reliable for describing the TEA molecule. The other basis sets and density functionals are included to ensure that trends in the dimer frequencies are consistent.

### DVR-Based Frequency Decomposition Analysis

The goal of a vibrational DVR calculation is to construct the Hamiltonian matrix, ***H***, for a particular vibration in a grid point position basis and then diagonalize it. The grid is formed along a vibrational coordinate, *Q*, with *P* points *Q*_1_, *Q*_2_, *Q*_3_,···*Q*_*P*_ equally spaced by Δ*Q*. The elements of the Hamiltonian matrix are given by,

1 $$\begin{array}{l} H_{\mathrm{ij}}=T_{\mathrm{ij}}+V\left(Q_{i}\right)\delta_{\mathrm{ij}} \end{array}$$

The values *i* and *j* index the grid points from 1 to the total number of grid points *P*, which in this case was always 20. *V*(*Q*) is the Born–Oppenheimer PES for the vibration, which is a function of the vibrational coordinate *Q*. The elements of the kinetic energy operator, ***T***, have previously been found based on a sinc-function basis set.^14,21^ Once the Hamiltonian matrix is constructed, it can be diagonalized. The vibrational frequency we are interested in can be found by taking the difference between the two lowest eigenvalues.

To determine the effect of charge transfer, polarization, and frozen orbital interactions on the vibrational frequencies, we manipulated the PESs used in our DVR method. For each molecular geometry composing a vibrational grid, we apply energy decomposition analysis based on absolutely localized molecular orbitals (ALMO).^22^ The terminal alkyne molecule (PAC or EBA) was treated as one fragment and the TEA molecule was treated as another; each fragment was assumed to be neutrally charged. The ALMO calculations were performed across the vibrational coordinate, allowing us to decompose the full vibrational PES into terms representing the potential energy of the isolated molecules (ISO), electrostatics (ELEC), Pauli repulsion (PAULI), dispersion (DISP), the polarization energy (POL), and charge transfer (CT)

2 $$\begin{array}{l} V\left(Q\right)=V_{\mathrm{ISO}}\left(Q\right)+\Delta E_{\mathrm{ELEC}}\left(Q\right)+\Delta E_{\mathrm{PAULI}}\left(Q\right)+\Delta E_{\mathrm{DISP}}\left(Q\right)+\Delta E_{\mathrm{POL}}\left(Q\right)+\Delta E_{\mathrm{CT}}\left(Q\right) \end{array}$$

The isolated potential energy, *V*_ISO_(*Q*), is the energy the system would have if the fragments were infinitely separated. The frozen orbital interaction, Δ*E*_FRZ_(*Q*) = Δ*E*_ELEC_(*Q*) + Δ*E*_PAULI_(*Q*) + Δ*E*_DISP_(*Q*), describes the energy change upon bringing the molecules together without allowing the molecular orbitals to change from their isolated configurations. This energy term can be broken down into contributions from permanent electrostatics, Δ*E*_ELEC_(*Q*), Pauli repulsion, Δ*E*_PAULI_(*Q*), and dispersion, Δ*E*_DISP_(*Q*). The polarization energy contribution, Δ*E*_POL_(*Q*), arises from the *intramolecular* relaxation of the orbitals of each molecule in the presence of the other molecule and its orbitals. Finally, the charge transfer energy, Δ*E*_CT_(*Q*), describes the energy change when the orbitals are allowed to mix between the fragments and so the full Born–Oppenheimer potential energy surface, *V*(*Q*), is recovered.^23^ These interaction energies can be incorporated into the DVR procedure described above to obtain the effect of these interactions on the vibrational frequencies. First, we construct a Hamiltonian where a selected effect has been removed

3 $$\begin{array}{l} H_{m‐\mathrm{REM},\mathrm{ij}}=T_{\mathrm{ij}}+\left[V_{\mathrm{ISO}}\left(Q_{i}\right)+\sum_{n\neq m}\Delta E_{n}\left(Q_{i}\right)\right]\delta_{\mathrm{ij}}\to \omega_{m‐\mathrm{REM}} \end{array}$$

where *m* is the interaction that we would like to remove and *n* iterates over all the ALMO interactions. When this Hamiltonian is diagonalized, we obtain the frequency that would be obtained if interaction *m* were not present, ω_*m*‑REM_. This frequency can then be compared to the frequency obtained when all effects are included in the Hamiltonian, ω_FULL_, to obtain the frequency change due to *m*, Δω_*m*_,

4 $$\begin{array}{l} H_{\mathrm{FULL},\mathrm{ij}}=T_{\mathrm{ij}}+\left[V_{\mathrm{ISO}}\left(Q_{i}\right)+\sum_{n}\Delta E_{n}\left(Q_{i}\right)\right]\delta_{\mathrm{ij}}\to \omega_{\mathrm{FULL}} \end{array}$$

5 $$\begin{array}{l} \Delta \omega_{m}=\omega_{\mathrm{FULL}}-\omega_{m‐\mathrm{REM}} \end{array}$$

This method uses the ALMO framework to decompose the effects on the frequency into understandable terms, so we call it ALMO- frequency decomposition analysis (ALMO-FDA).

### Isotropic Transition Polarizabilities

The DVR method provides vibrational wave functions which can be used to compute matrix elements of any operator which can be defined and computed along the normal mode coordinate, *Q*. The transition polarizability α_01_ is defined as the matrix element of the polarizability operator with the ground and first excited vibrational states, given by

6 $$\begin{array}{l} \alpha_{01}=\left\langle \Psi_{0}\left|\mathrm{\alpha ̂}\left(Q\right)\right|\Psi_{1}\right\rangle \end{array}$$

where *α̂*(*Q*) is the trace of the polarizability tensor surface along the normal mode coordinate, *Q*,

7 $$\begin{array}{l} \mathrm{\alpha ̂}\left(Q\right)=\frac{{\mathrm{\alpha ̂}}_{\mathrm{xx}}\left(Q\right)+{\mathrm{\alpha ̂}}_{\mathrm{yy}}\left(Q\right)+{\mathrm{\alpha ̂}}_{\mathrm{zz}}\left(Q\right)}{3} \end{array}$$

The polarizability matrix elements are calculated according to the finite field approach in Q-Chem. α_01_ is directly related to the intensity of Raman scattering which is observed in experiments.^24,25^

### Molecular Dynamics Simulations

All molecular dynamics (MD) simulations were performed using OpenMM version 7.6.^26^ The pressure in NPT simulations was kept constant using a Monte Carlo barostat adjusting the box volume isotropically every 25 timesteps, and the temperature in NPT and NVT simulations was held constant using the Nose-Hoover algorithm with a frequency for interactions with the heat bath of 1 ps^–1^.^27,28^ Initial simulation structures were obtained using PACKMOL.^29^ The cubic and periodic simulation boxes were packed to near the experimental density of TEA, the solvent molecule. The lengths of all bonds involving hydrogen was held constant, allowing a time step of 2 fs. The dispersive and electrostatic cutoff in our simulations was 1.4 nm and particle-mesh Ewald summation was used to correct long-range electrostatics.^30^ Because we were using the OPLS force field, Lennard-Jones parameters between unlike molecules were found using the geometric mean, i.e., $\sigma_{\mathrm{ij}}=\sqrt{\sigma_{\mathrm{ii}}\sigma_{\mathrm{jj}}}$ ; $\varepsilon_{\mathrm{ij}}=\sqrt{\varepsilon_{\mathrm{ii}}\varepsilon_{\mathrm{jj}}}$ .^31^ Unless otherwise mentioned, the following equilibration procedure was used for all simulations: (1) energy minimization to a maximum energy of 10 kJ mol^–1^, (2) random initialization of atomic velocities according to the Maxwell–Boltzmann distribution at 5 K, (3) temperature increase from 5 to 300 K in 1000 steps over 1 ns as pressure is held constant at 1 bar, (4) equilibration under NPT (300 K, 1 bar) conditions for 1 ns, (5) equilibration under NVT (300 K) conditions for 1 ns. Systems were considered to have equilibrated if their volume (in the NPT step) and if their total energy (in the NVT step) were generally constant in the last 0.5 ns of the appropriate step.

The OPLS-AA force field was used for these simulations, including the alkyne parameters from Rego et al.^31,32^ LigParGen was used to generate an initial OPLS force field for each molecule.^33^ The triethylamine parameters were used without adjustment, and result in a solvent density within 4% of the experimental value. The alkyne parameters were adjusted in some cases to match Rego et al. For PAC, the terminal alkyne angle strengths for C04–C05–C06 and C05–C06–H0C were adjusted to the values found by Rego et al. (Figure 1). The H0C, C06, and C05 Lennard-Jones parameters were also adjusted. Because of the aromatic ring in EBA, we were more careful in our adjustment - this moiety was not included in the set of molecules parametrized by Rego et al. The bonding parameters for the C08–C05 interaction given by LigParGen were similar to those from Rego et al., but the LigParGen parameters described a slightly shorter (by ∼0.002 nm) and stronger (by ∼8000 kJ mol^–1^ nm^–2^) bond. We believe the LigParGen parameters are more accurate in this case, since the aromatic ring would likely have the effect of strengthening the triple bond through resonance effects. Similar angle and Lennard-Jones parameters were adjusted as in the PAC case, except that the Lennard-Jones parameters for C09 were not changed from LigParGen’s estimate. The LigParGen and Rego et al. parameters for C09 differed by less than 0.02 nm for σ and 0.03 kJ mol^–1^ for ε. Partial charges for the alkynes were obtained from a ChElPG analysis of each molecule using PBE0/6-31G­(d,p) in Q-Chem as recommended by Rego et al.^15,17,32,34,35^ Parameters not mentioned in this paragraph for EBA and PAC were identical between Rego et al. and LigParGen, or were not given in Rego et al.

**Figure 1:**
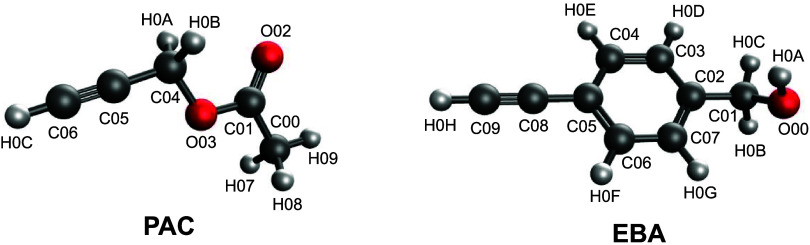
Atom name assignments for PAC and EBA. For PAC (EBA), H0C (H0H) is the terminal hydrogen, C06 and C05 (C09 and C08) are the triple bonded carbons, and C04 (C05) is the first R group carbon.

Four simulation boxes were created, each consisting of a single alkyne molecule (EBA or PAC) solvated in TEA. The initial simulation boxes were created using PACKMOL to a size of 4.2 nm × 4.2 nm × 4.2.^29^ The number of TEA molecules added was roughly set to match the experimental density of TEA at 300 K, minus one molecule to make room for the alkyne. Once equilibrated simulation boxes were obtained, production simulations were performed for an additional 200 ns, collecting snapshots every 2 ps for a total of 100,000 snapshots. Simulation boxes with initial dimensions of 6.2 nm × 6.2 nm × 6.2 nm were also created and equilibrated, then run for 20 ns. 1000 snapshots were collected from these simulations with a time separation of 20 ps for spectroscopic mapping and DFT analysis. Finally, additional 25 ns simulations with initial box dimensions of 6.2 nm × 6.2 nm × 6.2 nm were run where snapshots were collected every 0.1 ps in order to calculate Raman spectra.

### Cylindrical Distribution Function

While the commonly used radial distribution function provides a great deal of useful information on the solvent environment experienced by an atom, it suffers from an important limitation in this case. The terminal alkyne has cylindrical symmetry but the radial coordinate is spherically symmetric. This means that the radial distribution function averages together very different environments from the point of view of the alkyne. A cylindrical distribution function (CDF) uses two coordinates and can engage more fruitfully with the alkyne’s shape. To define these coordinates, we took the location of the carbon in the triple bond connected to the R group as the origin of the coordinate system. One coordinate unit vector, *ẑ*, was the unit vector in the direction connecting this carbon to the other triple bonded carbon. This coordinate can be positive or negative. The other coordinate unit vector, *r̂*, was the radius of a circle in the plane orthogonal to *ẑ* with the same origin. This coordinate can only be positive. Using these vectors, a CDF, *g*(*r*, *z*), can be defined as,

8 $$\begin{array}{l} g\left(r,z\right)=\frac{\left\langle \rho \left(r,z\right)\right\rangle }{\rho }=\frac{\left\langle N\left(r,z\right)\right\rangle }{\pi \left(2r\Delta r^{2}+\Delta r^{3}\right)\rho } \end{array}$$

Where z and *r* are the CDF coordinates of an atom of interest, ρ­(*r*, *z*) and *N*(*r*, *z*) are the density and number of atoms of interest at that location, respectively, ρ is the density of atoms of interest throughout the simulation, and Δ*r* = Δ*z* is the bin width.^36–38^ ⟨···⟩ indicates an average over all snapshots, counting atoms of interest within $r-\frac{\Delta r}{2}<r<r+\frac{\Delta r}{2}$ and $z-\frac{\Delta z}{2}<z<z+\frac{\Delta z}{2}$ . When *g*(*r*,*z*) = 1, then the density at the indicated location is the same as it would be if the alkyne was not in solution. As both *r* → *∞* and *z* → *∞*, we should expect *g*(*r*,*z*) → 1 since the alkyne will have weak effects on distant parts of the solution.

### Optimization of QM/MM Clusters

We selected 1000 snapshots from our larger 6.2 nm × 6.2 nm × 6.2 nm simulation box, separated by 20 ps each. This time separation was chosen to minimize frequency correlations between the subselected snapshots, based on experimental estimates of the frequency correlation time in similar solvents. We find that the correlation between adjacent frequencies separated by 20 ps is quite small, less than 0.04 in absolute value. The alkyne was centered in the box, and all TEA molecules with centers of mass more than 3 nm away from the alkyne hydrogen were removed. The alkyne molecule and the nearest TEA molecule to the terminal hydrogen were included in a “quantum mechanical” (QM) region, and all other TEA molecules were included in a “molecular mechanical” (MM) region. Using Q-Chem, atoms in QM molecules were treated using DFT and atoms in MM molecules were treated as point charges, with charges given by the OPLS molecular dynamics force field.

The maximum distance of TEA molecules included in the molecular mechanics region and the number of TEA molecules included in the quantum region was determined using 10 snapshots separated by 1 ns each (Figure 2). Initially, all TEA molecules with centers of mass less than 3 nm away from the alkyne hydrogen were included in the MM region. To optimize the quantum region, we used our LNM-DVR method to compute the vibrational frequency when 0 through 5 TEA molecules were included in the QM region. These frequencies were averaged across the 10 snapshots, then fit to the function ω = *c*–*be* ^–*N*/*a*^. Extrapolating, this function predicts that a frequency of *c* would be obtained if infinite QM TEA molecules were included. The majority of the frequency shift between 0 and 5 QM TEA molecules is captured using 1 QM TEA molecule, and the frequency obtained 5 QM TEA molecules is indistinguishable from the extrapolated frequency from an infinite number of QM TEA molecules. The difference between 5 QM TEA molecules and 1 QM TEA molecule was 3.3 (4.0) cm^–1^ for PAC (EBA). These values are added the final spectroscopic map to correct for additional solvent effects. Using this fixed QM region size of 1 TEA, we then reduced the maximum distance of TEA molecules included in the MM region. The frequency did not change even when the MM region was reduced to 1 nm, indicating that the 3 nm radius was converged. Because of the low cost of including MM TEA molecules, we used a maximum 3 nm cutoff for the MM region.

**Figure 2:**
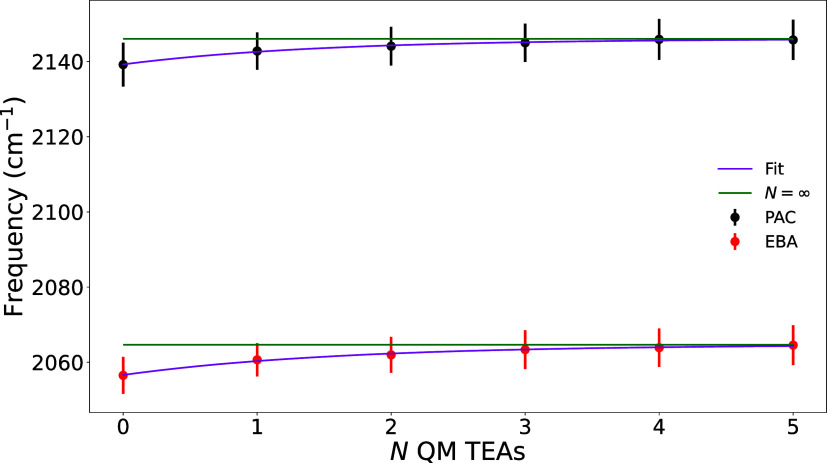
Convergence of frequency with respect to the number of QM TEA molecules. Error bars are 95% confidence intervals across 10 snapshots separated by 1 ns. Average frequencies were fit to the function ω = *c*–*be* ^–*N*/*a*^, where *N* is the number of QM TEA molecules.

### Molecular Effects on Frequency

The vibrational frequency from any individual snapshot can be broken down as follows,

9 $$\begin{array}{l} \omega =\omega_{g}+\Delta \omega \left(\theta_{\mathrm{RCC}},\theta_{\mathrm{HCC}}\right)+\Delta \omega_{R}+\Delta \omega_{\mathrm{TEA}} \end{array}$$

where ω_*g*_ is the gas phase frequency of the alkyne of interest, Δω­(θ_RCC_,θ_HCC_) is the change to the frequency arising from bends of the internal terminal alkyne angles (since the optimum angle is 180°, the dihedral only exists transiently), Δω_*R*_ is the change to the frequency arising from changes to other degrees of freedom in the alkyne, and Δω_TEA_ is the change to the frequency due to the TEA solvent. The effect of Δω_*R*_ on the experimental spectrum will likely be overestimated by frequency calculations performed on snapshots extracted from our MD simulations. This is mainly because these degrees of freedom will, in most cases, be in their vibrational ground state. This restriction is not present in the classical MD simulation so some unlikely configurations will be sampled more than they should be. Additionally, it is extremely difficult to build a spectroscopic map which includes the 3­(N-3)-6 additional degrees of freedom outside the terminal alkyne. To simplify, we assume that Δω_*R*_ = 0. This assumption will lead to an underestimate of the frequency distribution. Δω­(θ_RCC_,θ_HCC_) was obtained from calculations on gas phase structures of the alkynes, including constrained optimizations to specific pairs of angles. These data were fit to a smooth bivariate spline as provided in the scipy package and are available in the Supporting Information (SI).^39^

To obtain Δω_TEA_, we performed two sets of calculations on our MD snapshots. First, we computed the frequency of the terminal alkyne from the MD simulation with 1 QM TEA molecule and a 3 nm radius of MM TEA solvent, which we term ω_QM1MM3_. This can be broken down into contributions given by

10 $$\begin{array}{l} \omega_{\mathrm{QM}1\mathrm{MM}3}=\omega_{g}+\Delta \omega_{R}+\Delta \omega \left(\theta_{\mathrm{RCC}},\theta_{\mathrm{HCC}}\right)+\Delta \omega_{\mathrm{TEA}} \end{array}$$

Next, we computed the frequency for the same alkyne structures from the MD simulations, but with no QM or MM TEA molecules present. This is given the term ω_QM0MM0_ and can be broken down as

11 $$\begin{array}{l} \omega_{\mathrm{QM}0\mathrm{MM}0}=\omega_{g}+\Delta \omega_{R}+\Delta \omega \left(\theta_{\mathrm{RCC}},\theta_{\mathrm{HCC}}\right) \end{array}$$

Then, Δω_TEA_ is

12 $$\begin{array}{l} \Delta \omega_{\mathrm{TEA}}=\omega_{\mathrm{QM}1\mathrm{MM}3}-\omega_{\mathrm{QM}0\mathrm{MM}0} \end{array}$$

Because the alkyne structures are identical between the two calculations, ω_*g*_, Δω­(θ_RCC_,θ_HCC_), and Δω_*R*_ all cancel in this subtraction. Since we assume Δω_*R*_ = 0, and we can calculate ω_*g*_ and Δω­(θ_RCC_,θ_HCC_) separately, we can reconstruct a predicted frequency for any snapshot.

### Data for Spectroscopic Mapping

To develop a spectroscopic map, we ran a 20 ns simulation where we collected snapshots every 20 ps for each alkyne, for a total of 2000 snapshots. This collection frequency was determined based on the work of Londergan and co-workers.^8,12^ We then calculated Δω_TEA_ for each snapshot. To determine that 20 ps is sufficient to decorrelate the snapshots, we time ordered the frequencies and computed the correlation between snapshot frequency N and snapshot frequency N+1. This is equivalent to evaluating the frequency autocorrelation function with a time separation of 20 ps. For both alkynes the absolute value of the correlation between subsequent snapshots is less than 0.04. This data for Δω_TEA_ was split into a 1500-member training set and a 500-member test set. The alkynes were mixed together in these sets.

### Computational Raman Spectra

A higher resolution 25 ns simulation was run where snapshots were collected every 0.1 ps and frequencies were computed for each of these snapshots using the spectroscopic map developed in this work. The Raman spectrum was computed using the fluctuating frequency approximation

13 $$\begin{array}{l} I_{R}\left(\omega \right)\propto R\left[\int_{0}^{\infty }dt{\;e}^{i\omega t}\left\langle \alpha_{01}\left(t\right)\alpha_{01}\left(0\right)e^{i\int_{0}^{t}d\tau \delta \omega \left(\tau \right)}\right\rangle e^{-t/2T_{1}}\right] \end{array}$$

where α_01_(*t*) is the isotropic transition polarizability between the ground (0) and first excited (1) states at a particular time *t*, and *T*_1_ is the vibrational population lifetime which is estimated from experiment to be 5 ps.^40,41^ δω­(*t*) = ω­(*t*)–⟨ω⟩ tracks the instantaneous frequency fluctuations.

## Results and Discussion

### Molecular Dynamics Simulations

Molecular dynamics simulations of one terminal alkyne molecule (EBA or PAC) solvated in otherwise pure TEA were performed in cubic simulation boxes with an approximately 4 nm edge length. Cylindrical distribution functions (CDFs) were calculated and are shown in Figure 3.

**Figure 3:**
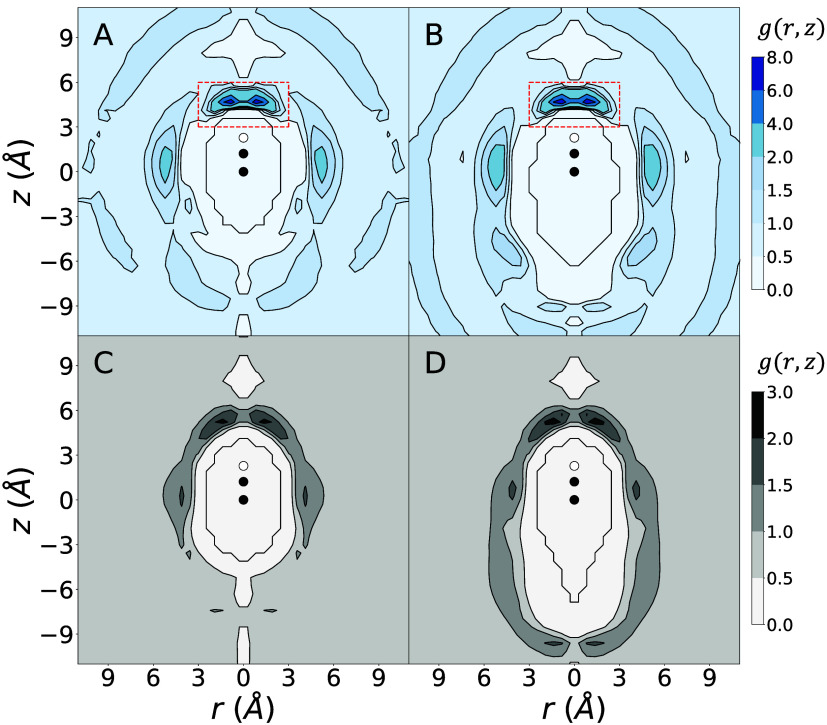
Cylindrical distribution functions for TEA nitrogen atoms (A and B) or carbon atoms (C and D) with respect to the terminal alkyne moiety of PAC (A and C) or EBA (B and D). The black circle at (0, 0) is the location of the carbon in the terminal alkyne bonded to the R group. This first carbon is triple bonded to the carbon represented by the black circle at about (0, 1.215). The terminal alkyne hydrogen is represented by a white circle at about (0, 2.276). The red box (A and B) highlights the main nitrogen peak. The *r* axis is reflected for clarity.

The effect of the substituent to the alkyne molecule on the structure of the solvent is relatively small, especially in the vicinity of the terminal alkyne moiety. There are some interactions between the triple bond and the TEA carbons, which cause peaks in both CDFs since the nitrogens are bound to the carbons. Most significantly, the CDFs clearly show a much stronger interaction between the terminal alkyne and the TEA nitrogen than between the terminal alkyne and the TEA carbons. There is also a small increase in density near the terminal alkyne hydrogen in the carbon CDFs. These are likely carbons which are bonded to the nitrogens which are directly interacting with the alkyne. The maximum CDF value observed for the nitrogen is about 7.3 for both alkynes, more than two times as much as is observed for the carbons. The largest nitrogen peak occurs near the terminal alkyne hydrogen. It is centered approximately 2 Å from the terminal alkyne hydrogen with an average C–H–N angle of 180°. It begins at approximately 1 Å from the terminal alkyne hydrogen and ends about 5 Å away. The peak is moderately spread out and the absolute maximum of the peak occurs away from its center. If we integrate this major peak between *z* = [3, 6] and *r* = [0, 3], (the area inside the red square in Figure 3) we find that it represents an average of 0.57 (0.61) TEA nitrogens near the terminal hydrogen of PAC (EBA). We can also count the number of nitrogen atoms in the same region over the course of our 200 ns simulation where snapshots were collected every 2 ps (Figure 4). For PAC (EBA) 59.0% (62.7%) of snapshots have exactly one nitrogen atom in the region of interest, 40.3% (36.4%) of snapshots have no nitrogen atoms in that volume, and 0.7% (0.8%) of snapshots have 2 nitrogen atoms. No snapshots have three or more nitrogens in the space (Figure 4). These values are nearly identical to those observed if we analyze our higher resolution 25 ns simulation where snapshots were collected every 0.1 ps. We say that the alkynes in snapshots where there is a nitrogen near the terminal alkyne hydrogen are “nitrogen associated.” If there is no TEA nitrogen near the hydrogen, the alkyne is said to be “carbon associated.” Because the number of snapshots where 2 or more TEA Ns are present is so small, the values from integration can be interpreted as a measure of the likelihood that the alkyne is nitrogen associated.

**Figure 4:**
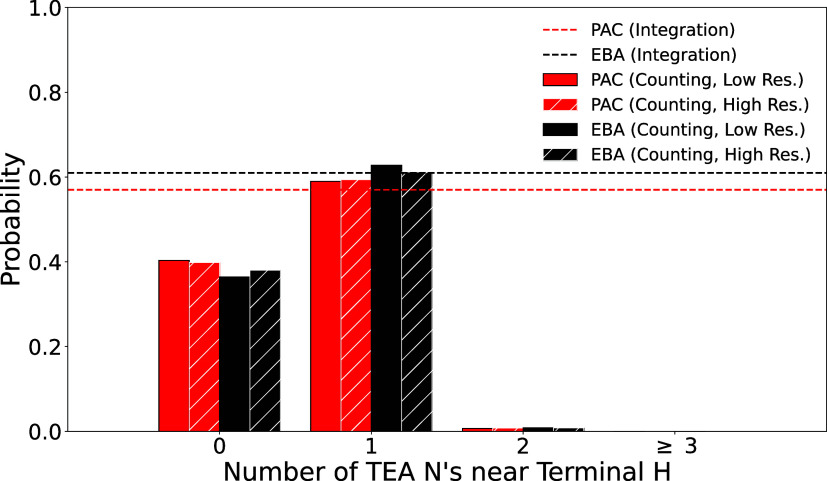
Probability of finding nitrogen atoms near the terminal alkyne hydrogen, taken by integration of the cylindrical distribution function, and by counting nitrogen atoms near the alkyne hydrogen in low-resolution (snapshots collected every 2 ps) and high-resolution (snapshots collected every 0.1 ps) simulations.

The fact that neither the sampling resolution nor the length of the simulation changes the apparent probability of nitrogen association for the terminal alkyne implies that the dynamical processes which change the number of nitrogens near the terminal alkyne are relatively slow, with time scales larger than 2 ps. We believe that the correct molecular picture is one where the alkyne spends significant time interacting with specific nitrogen atoms. After at least 2 ps, the interaction breaks and the alkyne does not interact with any nitrogen for a period of time. This is supported by the small number of cases where two nitrogens are near the terminal alkyne – it is unlikely that nitrogens “compete” to interact with the alkyne. To confirm this, we calculated the time correlation function for the number of nitrogens near the alkyne from our high-resolution simulation and fit it to a triexponential function. We then integrated the triexponential function to obtain the correlation time. For PAC (EBA), the correlation time was 3.1 ps (3.5 ps), and the longest time scale was 7.4 ps (9.1 ps). We interpret this to mean that the average time scale for alkyne-nitrogen interactions is about 3–4 ps, and the strongest interactions last about 7–10 ps.

### Dimer Quantum Mechanics

To understand the effect of the strong TEA nitrogen-alkyne hydrogen interaction on the vibrational frequency, we extracted one TEA-PAC dimer and one TEA-EBA dimer for further analysis using DFT and LNM-DVR based vibrational frequency calculations. The selected dimers initially exhibited nitrogen association (Figure 5). We performed constrained geometry optimizations where the alkyne hydrogen-TEA nitrogen distance (hereafter H–N distance) was constrained to increasing values and computed the terminal alkyne stretching frequencies of the resulting structures. Using ALMO calculations, we decomposed these frequencies into components due to Pauli repulsion, electrostatics, dispersion, polarization, and charge transfer.

**Figure 5:**
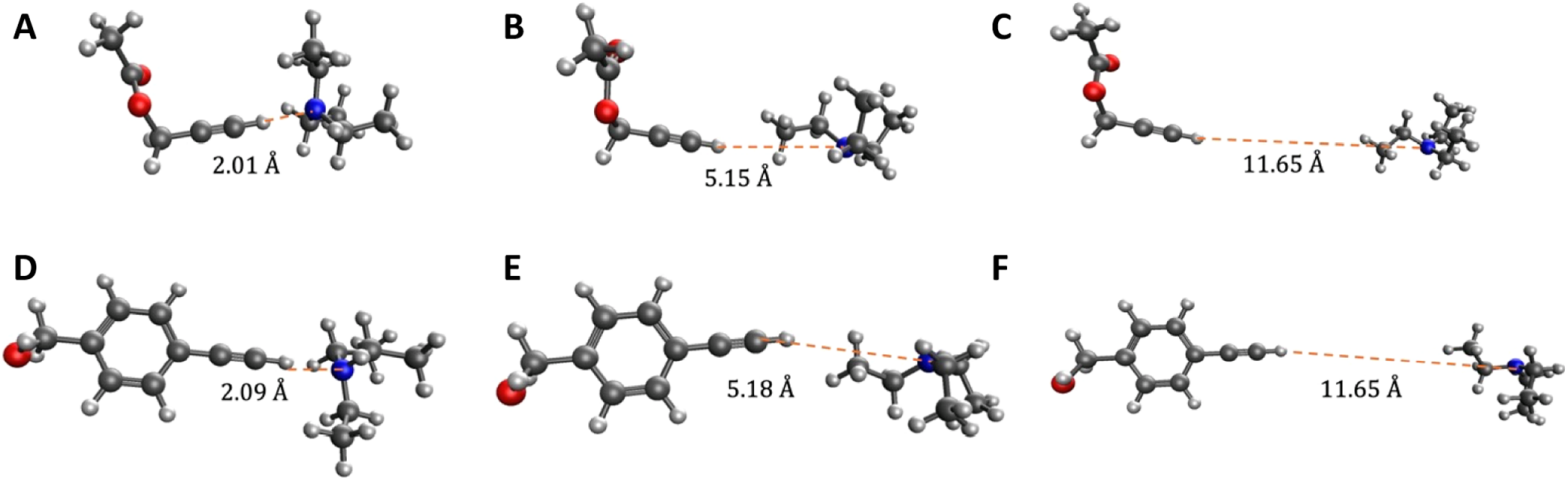
Optimized TEA-PAC (A–C) and TEA-EBA (D-F) dimers constrained to different H–N distances. (A and D) The fully optimized structures with no constraints. (B and E) Once there is enough space between the terminal alkyne and the TEA, ethyl groups fill the space and form carbon-associated structures. (C and F) At large distances, the TEA has no effect on the terminal alkyne.

In Figure 6, the frequency as a function of the H–N distance for three density functional methods are shown. The frequency is relatively low for nitrogen-associated structures, increasing as the distance between the H and N increases. After a H–N distance of about 5 Å, the TEA rotates such that an ethyl fills the space between the TEA and alkyne and the structure becomes carbon-associated. There is little change in the frequency as the H–N distance continues to increase from 5 to 12 Å, showing that a carbon-associated alkyne is similar to a gas phase alkyne. Multiple density functionals agree on this trend across both alkyne molecules. Due to this, we believe the TPSS-D3/6–311++G** method is reliable enough to be used exclusively for all remaining DFT calculations in this work.

**Figure 6:**
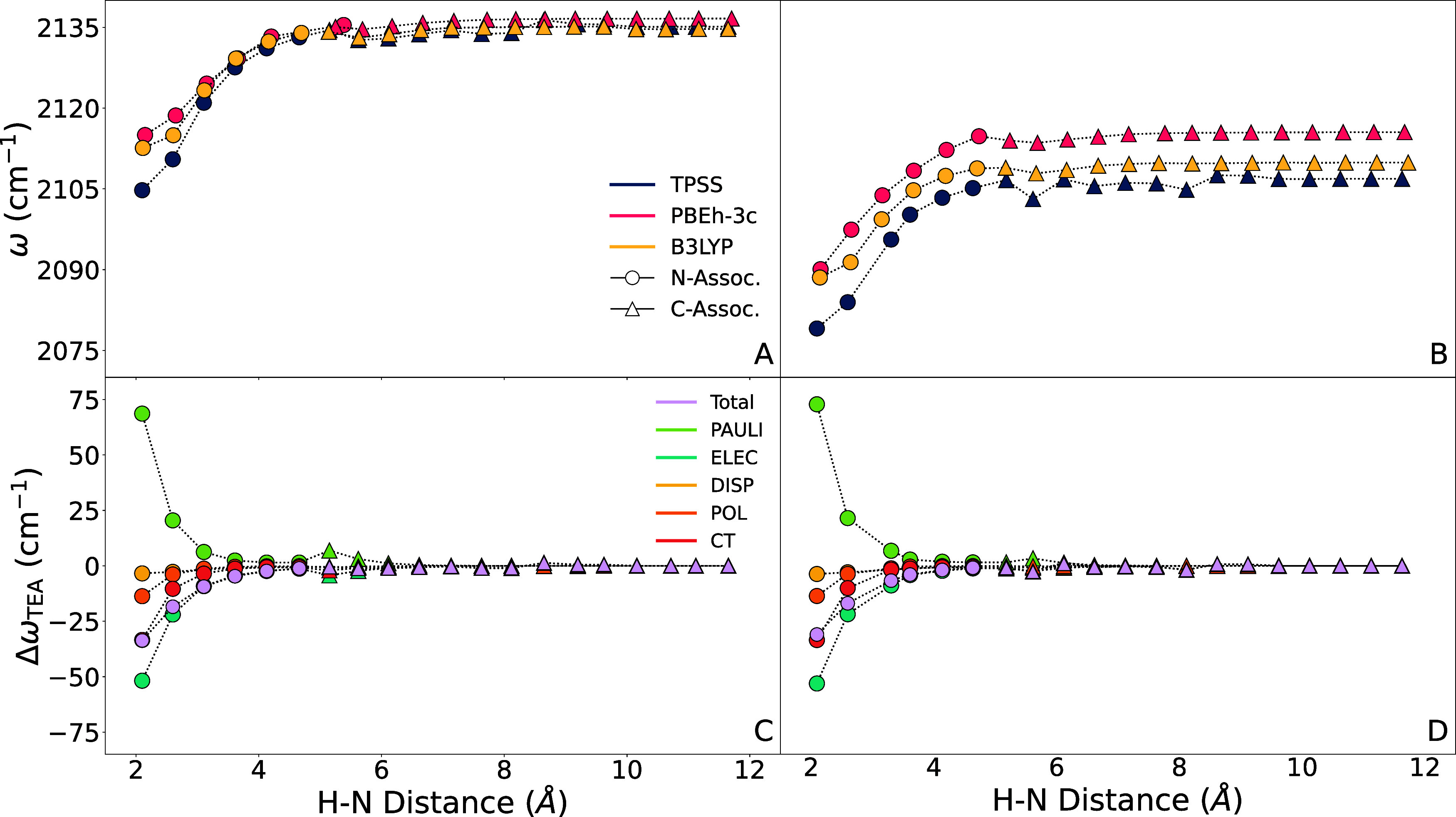
Frequency (A and B) and decomposition (C and D) as a function of the distance between the TEA nitrogen and the terminal alkyne hydrogen of PAC (A and C) or EBA (B and D). Frequencies (A and B) were calculated with three different methods: B3LYP-D3/def2-TZVPD, TPSS-D3/6–311++G**, and PBEh-3c/def2-mSVP. Decompositions were computed using TPSS-D3/6–311++G**.

Figure 6 also shows the decomposition of Δω_TEA_. For the nitrogen-associated structures, Pauli repulsion, charge transfer, and electrostatics have the largest effect on the frequency. Pauli repulsion tends to increase the frequency, while charge transfer and electrostatics tend to decrease it. The total frequency shift is determined by the balance of these effects – for these optimized structures, the effects which decrease the frequency sum to outweigh the frequency increase from Pauli repulsion. However, a spectroscopic map would need to be flexible enough to capture both of these frequency-increasing and frequency-decreasing effects, which might be differently balanced in an actual MD simulation.

In this system, both electrostatics and charge transfer can be understood to be related to the excess donatable electron density near the TEA nitrogen. In a nitrogen associated configuration, these electrons create a locus of negative charge near the partially positive terminal alkyne hydrogen (electrostatics) and interact favorably with the weakly Lewis acidic terminal alkyne hydrogen (charge transfer). Both of these effects tend to weaken the C–H bond and thus lower the frequency for the C≡C vibration. We would expect them to be strongest when the angle between the terminal alkyne carbon bonded to the terminal hydrogen, the terminal hydrogen, and the TEA nitrogen is 180°. The Pauli repulsion interaction is also related to the presence of electrons. However, we expect their strength to be less dependent on the angle between the alkyne and TEA than electrostatics or charge transfer.

### Spectroscopic Mapping

From our MD simulations, we know that the nitrogen from TEA interacts strongly with the terminal alkyne hydrogen about half the time. From our DFT calculations, we know that if the TEA nitrogen is interacting with the terminal alkyne hydrogen, then the frequency will decrease compared to carbon-association or the gas phase. How do these effects combine to produce the experimentally observed Raman spectrum?

First, we used 100 of our 1000 snapshots for each alkyne to evaluate the appropriateness of the Condon approximation that the scattering cross section of the vibration is uncorrelated with the vibrational frequency. We find it is valid – the calculated transition polarizabilities have no relationship to ω or to Δω_TEA_.

Next, we turned to determine good variables for a spectroscopic map. From our ALMO-FDA calculations, we have a good sense of the effects which alter the vibrational frequency of terminal alkynes interacting with TEA molecules – charge transfer, electrostatics, and Pauli repulsion. To build a spectroscopic map for the change in frequency due to the TEA, we need to find items in the MD force field which map well to these effects. Electrostatics and charge transfer may be captured by the electrostatics in the simulation – atoms with strong negative charges in the MD simulation tend to have larger local electron densities. To capture the directionality of these effects, we use the electric field rather than the electric potential. The damped shifted force method was used for these calculations.^42^ There is a weak correlation between Δω_TEA_ and the electric field due to TEA molecules at the site of the hydrogen atom and pointed in the direction of the C–H bond. We also find that there are similar correlations to the first and second derivatives of the electric field at the site of the terminal hydrogen in the direction of the C–H bond. Derivatives were found using the appropriate three-point centered finite difference approximations with a 0.1 Å spacing between points.

The Pauli repulsion effect is modeled in the OPLS force field by the repulsive portion of the Lennard-Jones potential. To capture this, we examined the repulsive part of the Lennard-Jones potential at each atom in the terminal alkyne moiety – the triple bonded carbons and the terminal hydrogen. The repulsions were calculated by summing the interactions of all non-nitrogen atoms – including the nitrogen atoms actually reduced the effectiveness of this variable. Most likely, the effects from the nitrogen atoms are already included in the electrostatic variables. By themselves, the correlations of each of these repulsive Lennard-Jones variables to Δω_TEA_ are small. However, when added through multilinear regression to the electric field derivatives, the overall correlation with Δω_TEA_ increases significantly. We also tested the attractive Lennard-Jones potentials, but the performance was worse or no better depending on exactly how these interactions were added to the model.

Our final map is based on multilinear regression of the variables described above using scikit-learn.^43^ As an equation, it is

14 $$\begin{array}{l} \omega_{\mathrm{MAP}}=\omega_{g}+\Delta \omega \left(\theta_{\mathrm{RCC}},\theta_{\mathrm{HCC}}\right)+\Delta \omega_{\mathrm{TEA}}+\Delta \omega_{\mathrm{QM}1\to \mathrm{QM}\infty } \end{array}$$

where

15 $$\begin{array}{l} \Delta \omega_{\mathrm{TEA}}=\sum_{k=0}^{2}a_{k}\frac{\partial^{k}({\mathrm{r̂}}_{CH}\cdot \left[\sum_{n=1}^{N}{\mathrm{E⃗}}_{\mathrm{Hn}}\right])}{\partial r_{CH}^{k}}+\sum_{i=1}^{3}\left(b_{i}\sum_{m=1}^{M}4\varepsilon_{m}{\left(\frac{\sigma_{m}}{r_{\mathrm{mi}}}\right)}^{12}\right) \end{array}$$

where *n* iterates over all *N* atoms, *E⃗*_*Hn*_ is the *n*th atomic contribution to the electric field at the terminal hydrogen, *r̂*_CH_ is the unit vector pointing across the C–H bond (assuming the C≡C–H angle is 180°), *r*_CH_ is the length of the C–H bond, and the *a*_*k*_ are coefficients found through multilinear regression for each order of derivative *k*. In the second term, *i* iterates over the three terminal alkyne atoms (C≡C–H), each of which has been assigned a coefficient *b*_*i*_ by multilinear regression. *m* iterates over all *M* non-nitrogen solvent atoms, ε_*m*_ and σ_*m*_ are the Lennard-Jones coefficients for that atom (no mixing rules were used) and *r*_*mi*_ is the distance between solvent atom *m* and terminal alkyne atom *i*. To obtain the complete frequency ω, we also add the gas phase frequency ω_*g*_ (2138.39 cm^–1^ for PAC and 2110.65 cm^–1^ for EBA), the angle effect Δω­(θ_RCC_,θ_HCC_) based on a smooth bivariate spline approximation to gas phase LNM-DVR calculations of each alkyne across the space of accessible angles (included in the SI), and the additional missing solvent effects captured by comparing our QM1 calculations to “infinite” QM extrapolations, Δω_QM1→QM∞_. The value of Δω_QM1→QM∞_ is 3.3 (4.0) cm^–1^ for PAC (EBA). The coefficient values are given in Table 1.

**Table 1:** Multilinear Regression Vibrational Spectroscopic Map Coefficients for the Frequency Change Due to the TEA Solvent^a^

**symbol**	**coefficient value**
*a*_0_	–1.51 × 10^2^ *E*_h_^–1^ *e a*_0_ cm^–1^
*a*_1_	1.96 × 10^3^ *E*_h_^–1^ *e a*_0_^2^ cm^–1^
*a*_2_	–1.06 × 10^3^ *E*_h_^–1^ *e a*_0_^3^ cm^–1^
*b*_H_	3.78 × 10^2^ *E*_h_^–1^ cm^–1^
*b*_CH_	3.0 × 10^2^ *E*_h_^–1^ cm^–1^
*b*_CR_	–1.49 × 10^3^ *E*_h_^–1^ cm^–1^

a H refers to the terminal hydrogen, CH refers to the triple bonded carbon also bound to the terminal hydrogen, and CR refers to the triple bonded carbon also bound to the R group. The coefficients were determined using a randomly selected 3/4 of the 2000 snapshots of both alkynes in TEA. Uncertainty in the coefficients was estimated by fitting 25 randomly selected 3/4 training sets and finding the 95% confidence interval of the mean of the coefficients found with these training sets. The final digit shown in each entry in the table below is uncertain. Because the units for each map variable are generally different, each coefficient is reported using the appropriate atomic units.

Table 2 shows the average and standard deviation change to the frequency for each variable. As expected, the electric field derivatives together tend to decrease the frequency. Interestingly, the electric field and its second derivative both generally increase the frequency. Also interesting is that, while the repulsive LJ energies at the terminal hydrogen and the carbon bonded to it increase the frequency, the same variable decreases the frequency at the other triple bonded carbon.

**Table 2:** Contributions to the Overall Vibrational Frequency of Each Map Variable for 500,000 Snapshots^a^

**coefficient symbol**	**⟨Δω⟩**	**σ**_**Δω**_	**N-assoc ⟨Δω⟩**	**N-assoc** ***σ***_**Δω**_	**C-assoc ⟨Δω⟩**	**C-assoc** **σ**_**Δω**_
*a*_0_	0.81	0.93	1.32	0.84	0.01	0.22
*a*_1_	–4.20	6.23	–6.89	6.61	0.03	1.43
*a*_2_	0.72	2.12	1.25	2.54	–0.11	0.49
*b*_H_	2.25	1.83	2.76	1.92	1.46	1.32
*b*_CH_	0.35	0.16	0.36	0.16	0.32	0.15
*b*_CR_	–0.97	0.48	–0.98	0.48	–0.92	0.45
total	–1.04	3.30	–2.18	3.50	0.75	1.86

a ⟨Δω⟩ refers to the average contribution, and σ_Δω_ is the associated standard deviation. The contribution of each variable in only nitrogen associated and carbon associated snapshots is also presented. All values are in wavenumbers, cm^–1^, and include data from both alkynes.

Breaking the statistics into a nitrogen associated case and a carbon associated case clarifies the situation. Two items become apparent. First, the LJ variables associated with the triple bonded carbons do not change much between the two environments. This likely means they mainly describe the effects of the solvent, beyond the nearest TEA, on the frequency. This effect was not present in the ALMO-FDA calculations described above. The repulsive potential on the terminal hydrogen does change somewhat between the two environments – the portion that changes likely reports on Pauli repulsion effects in the N-associated case.

Second, the electric field variables contribute nearly nothing to the frequency in the C-associated case. This means they must be entirely reporting on effects present in the ALMO-FDA calculations associated with the nearest TEA. Focusing on the N-associated case, the electric field always contributes a positive change to the frequency, so it must in part report on Pauli repulsion effects in addition to a small portion of the electrostatics. It is important to remember that the electrostatics present in the MD force field are a subtly different quantity than the electrostatics obtained from the ALMO analysis. The electrostatics present in the MD force field combine the effects of charge transfer, polarization, and permanent electrostatics that are carefully separated in the ALMO approach. We should not necessarily expect a 1-to-1 correlation between MD electrostatics and ALMO electrostatics.

The first derivative of the electric field has a large negative contribution, so we assign it to a combination of charge transfer, polarization, and electrostatics. The second derivative has a positive mean contribution, but the standard deviation is large enough that it provides a negative instantaneous contribution in many cases. Since we would expect it to report on short-range interactions, we assign this variable to a combination of Pauli repulsion and charge transfer – qualitatively, this variable is capturing the movement of electrons between the terminal alkyne and the TEA.

Evaluated against our 500-member two-alkyne test set, this map had a correlation coefficient of 0.82 with a root mean squared error of 2.44 cm^–1^ (Figure 7). Similar statistics and coefficients are obtained if maps are created and tested with one-alkyne data sets instead of two-alkyne data sets. We tested several other types of regression model including artificial neural networks. All performed no better than multilinear regression. Artificial neural networks which were too small struggled to learn anything from the training set; networks which were only slightly larger tended to provide identical performance to multilinear regression.

**Figure 7:**
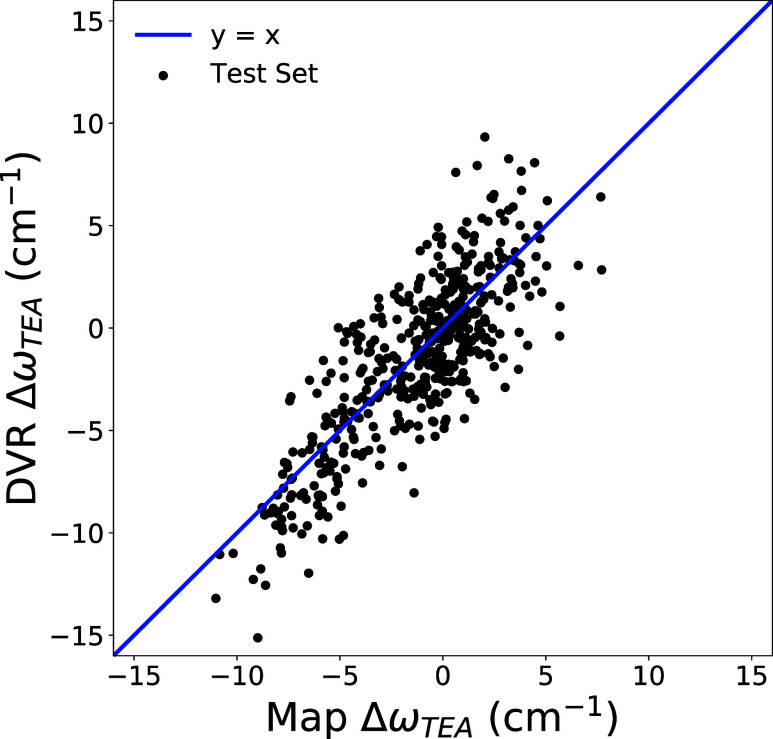
Performance of the spectroscopic map developed in this work to compute the effect of the solvent on the alkyne probe frequency. The blue line represents a perfect correlation. The correlation between map frequencies and DVR frequencies in the test set is 0.82 and the root-mean-squared-difference is 2.44 cm^–1^.

### Computational Raman Spectra

With this map in hand, we performed MD simulations of each alkyne in TEA for 25 ns where snapshots were collected every 0.1 ps. The map variables were extracted from this simulation and map frequencies were computed for every snapshot. This frequency trajectory was used to compute Raman spectra according to the fluctuating frequency approximation (eq 13). These Raman spectra and the underlying frequency distributions are shown in Figure 8.

**Figure 8:**
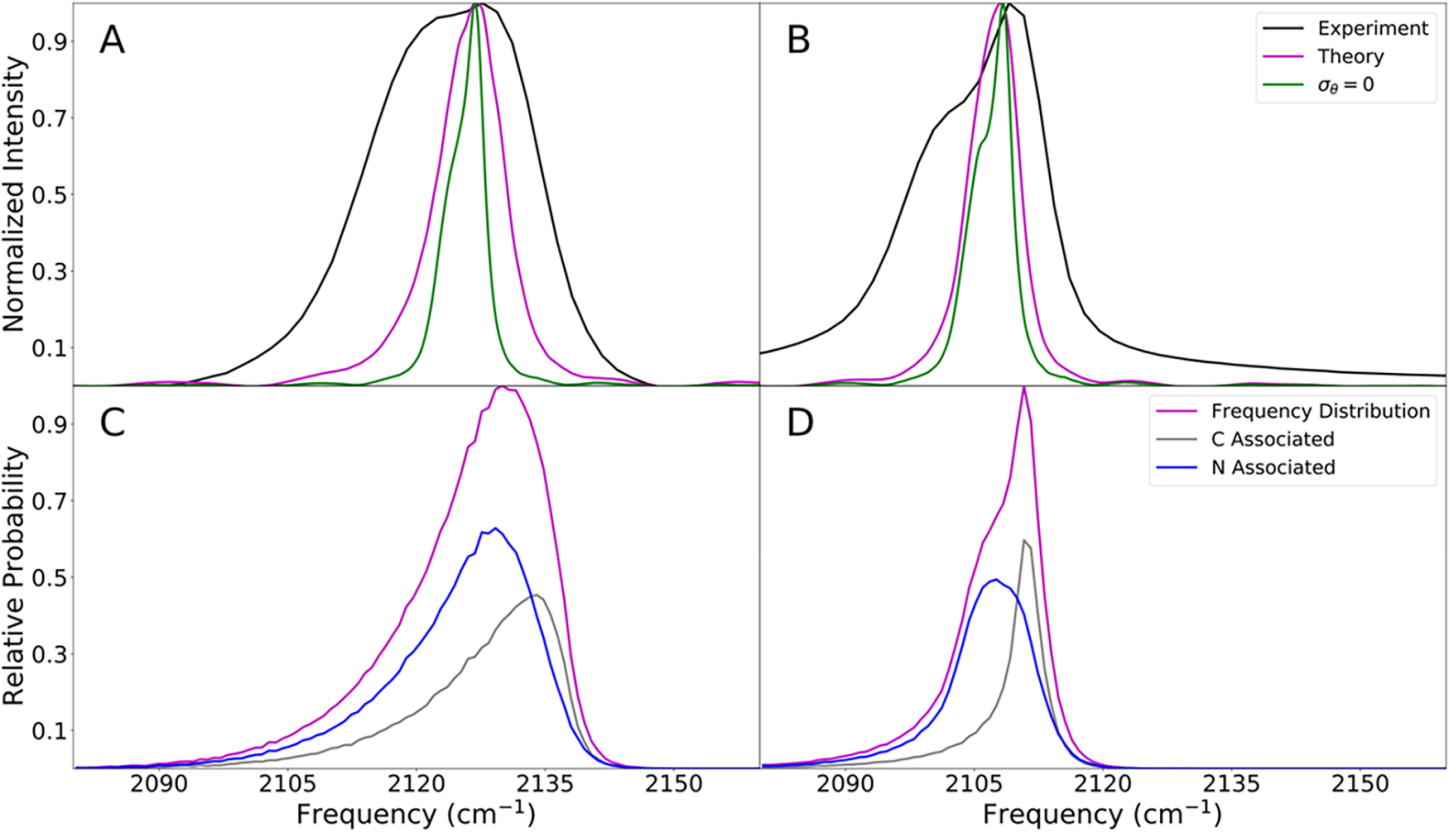
Raman spectra and frequency distributions computed using the spectroscopic map for PAC (A and C) and EBA (B and D). (A and B) Experimental data from ref 8 and predicted Raman spectra. The green curve is the predicted Raman spectrum calculated under the condition that the angle contribution to the frequency is zero. (C and D) The computational frequency histograms, broken down into nitrogen associated and carbon associated populations.

One of the most interesting features of the experimental spectra is their pronounced asymmetry. They are each fit well by a pair of Gaussian functions, implying bimodal behavior. Our spectra are also asymmetric. In particular, they are broadened to the left, like the experimental spectra. To determine the origin of the asymmetry in the computed spectra, we examine the underlying frequency distributions. These are also asymmetric, and this asymmetry arises for two reasons. First, bending of the CCH and CCC angles of the alkyne tend to reduce the frequency. However, this likely does not explain the experimental asymmetry. This angle effect would show up in the experiment as a hot band at lower frequencies, and the intensity of this band would increase with temperature. However, the experiments show a decrease or no change in the left side of the spectrum with an increase in temperature, so the hot band is likely at a much lower frequency than the main band.^8^ In addition, a version of the simulated spectra where frequency variance due to the angle is removed is still asymmetric. In fact, this version of the computational spectra appear clearly bimodal, like the experimental spectra. The underlying frequency distribution has been broken down into nitrogen-associated and carbon-associated populations in Figure 8C,D. For both alkynes, the nitrogen-associated population has a lower average frequency. When put together with the carbon associated population, we obtain a bimodal distribution as is seen in the Raman spectra. This is consistent with experiments which see lower terminal alkyne vibrational frequencies when the terminal alkyne has access to Lewis bases and bimodal Raman spectra when the terminal alkyne is present in high and low Lewis basic environments.^8,11,44^

Our computed Raman spectra are significantly narrower than experiment. There are several possible reasons for this. First, the DFT calculations may be inaccurate, returning similar frequencies for different snapshots. However, we see in Figure 5 that the frequencies returned by distinct DFT methods agree strongly with each other, implying that the TPSS results are accurate. Second, the spectroscopic map may be missing variance which is present in the DFT calculations. This is again unlikely, since the correlation coefficient for the map is relatively high and the RMSD is small. Another possibility is that the spectrum has been narrowed through incorrect dynamical effects introduced by inaccuracies in our MD simulations. If this were the case and there were no other errors, the underlying frequency distribution width calculated with our map would be similar to the experimental width, but also larger than the calculated spectrum width. We do not find this to be true – the experimental width is much greater than the map frequency distribution width (Table 3). The most likely explanation is that the missing width in the experimental Raman spectrum is due to the assumption that Δω_*R*_ = 0. When this assumption is not made, as in the distribution of the 1000 raw ω_QM1MM3_ frequencies, the fwhm of the frequency distribution is actually larger than the experimental value, while the peak frequency is less accurate. Unfortunately, it currently infeasible to develop a map which captures all of the degrees of freedom of arbitrary R groups. Even so, the current map allows for the calculation of all remaining features of the Raman spectra.

**Table 3:** Peaks and Full Width at Half-Maximum (FWHM) for Experimental^8^ Raman Spectrum, Computed Raman Spectrum, *I*_*R*_(ω), Map Frequency Distribution, ω_MAP_, and the Frequency Distribution for the 1000 ω_QM1MM3_ Frequencies Where Δω_*R*_ ≠ 0^a^

**distribution**	**PAC peak**	**PAC fwhm**	**EBA peak**	**EBA fwhm**
experiment	2127.7	22.1	2111.7	15.5
*I*_*R*_(ω)	2127.0	8.6	2108.1	6.7
ω_MAP_	2130.5	16.0	2110.8	8.0
ω_QM1MM3_	2141.0	24.0	2058.0	20.0

a Values are presented for each of the two investigated alkynes.

## Conclusions

Together, this computational and experimental evidence clearly shows that association with the electron dense nitrogen in TEA creates a population of low frequency terminal alkynes which show up in the experimental spectrum as a shoulder. We predict that these associations are relatively long lasting, on the order of 3–10 ps. This is similar to the persistence of hydrogen bonds in water.^45^ The overall free energy for the terminal alkyne existing in the electron rich and electron poor environments is nearly equal, with a small preference for the electron rich environment. However, the frequency is less variant in the electron poor environment, leading to a sharper peak in that population. Our results imply that the center frequency of the alkynes reports on the availability of such electron rich environments. In future work, we will extend this analysis and our spectroscopic map to additional solvent environments. We also find that our computational spectra are narrower than experiment, likely because we have ignored the R group effects which are difficult to build a map for. These R group effects could hypothetically be modeled by a random process with the appropriate correlation time in future work. Example code for the LNM-DVR method, snapshot extraction, calculation of map variables, and Raman spectrum calculations are given at https://github.com/Daly-Lab-at-Haverford/code_examples.

## Supplementary Material



## Notes

A text file containing the gas phase frequency as a function of the CCH and RCC angles needed to generate the spline for Δω­(θ_RCC_,θ_HCC_) using scipy.interpolate.SmoothBivariateSpline­() (TXT)
